# Cisplatin, environmental metals, and cardiovascular disease: an urgent need to understand underlying mechanisms

**DOI:** 10.1186/s40959-021-00120-z

**Published:** 2021-10-10

**Authors:** Suparna C. Clasen, Paul C. Dinh, Lifang Hou, Chunkit Fung, Howard D. Sesso, Lois B. Travis

**Affiliations:** 1 Krannert Institute of Cardiology, Department of Medicine, Indiana University School of Medicine, Indiana University, 1800 N. Capitol Ave, E308, Indianapolis, IN 46202 USA; 2 Division of Hematology-Oncology, Department of Medicine, Indiana University School of Medicine, Indianapolis, IN USA; 3 Center for Global Oncology Institute for Global Health, Feinberg School of Medicine, Northwestern University, Chicago, IL USA; 4 Division of Hematology and Oncology, Department of Medicine, University of Rochester School of Medicine and Dentistry, James P. Wilmot Cancer Institute, Rochester, NY USA; 5 Division of Preventive Medicine, Department of Medicine, Brigham and Women’s Hospital and Harvard Medical School, Boston, MA USA; 6 Department of Epidemiology, Fairbanks School of Public Health, Indianapolis, IN USA

**Keywords:** Cisplatin, Testicular cancer, Survivorship, Vascular toxicity, Thrombosis, Heavy-metals

## Abstract

Significantly increased risks of cardiovascular disease occur in testicular cancer survivors given cisplatin-based chemotherapy. The postulated mechanism of platinum-based chemotherapy’s vascular toxicity has been thought secondary to its different early- and late- effects on vascular injury, endothelial dysfunction, and induction of a hypercoagulable state. We highlight for the first time the similarities between platinum-associated vascular adverse events and the vascular toxicity associated with other xenobiotic-metal contaminants. The vascular toxicity seen in large epidemiologic studies of testicular cancer survivors may in part be similar and mechanistically linked to the risk seen in environmental heavy metal contaminants linked to cardiovascular disease. Future research should be directed to better understand the magnitude of the adverse cardiovascular effects of platinum and to elucidate the underlying mechanisms of action.

## Short communication

Significantly increased risks of cardiovascular disease (CVD) occur in testicular cancer survivors (TCS) given platinum-based chemotherapy regimens such as bleomycin, etoposide, and cisplatin (BEP) [1–3].The postulated mechanism of platinum-based chemotherapy’s vascular toxicity has been thought secondary to its different early- and late- effects on vascular injury, endothelial dysfunction, and induction of a hypercoagulable state [2, 3]. Early, in the first year after therapy, TCS treated with platinum-based chemotherapy have markedly increased six-fold risks of myocardial infarction [1] and increased risks of cardiovascular mortality [2]. Further, there are also early increased risks of cerebrovascular accidents [1, 2], vascular thromboembolism [1], and new-onset hypertension [1]. Early adverse vascular effects of platinum drugs, occurring less than one year after exposure, are thought to be due to acute vasospasm (days to weeks after exposure) and acute thrombosis (weeks to months after exposure) [4], which are mechanistically linked to disruptions in vascular smooth muscle cells and endothelial cells [3].

There are also elevated risks of late, occurring typically more than ten years after treatment, atherosclerotic CVD events after platinum-based chemotherapy, including an increased risk of myocardial infarction (HR, 1.4; 95% CI, 1.0–2.0) and cardiovascular death (HR, 1.6; 95% CI, 1.0–2.5) [1]. Other late risks following BEP include the development of hypertension, hyperlipidemia, diabetes mellitus, and metabolic syndrome [1, 2]. Late adverse vascular effects of platinum drugs have been attributed to platinum-related disruptions of vascular endothelial cells [3] resulting in a proinflammatory state, leading to atherosclerosis (occurring months to years after exposure), and “accelerated vascular aging” [5].

**Fig. 1:**
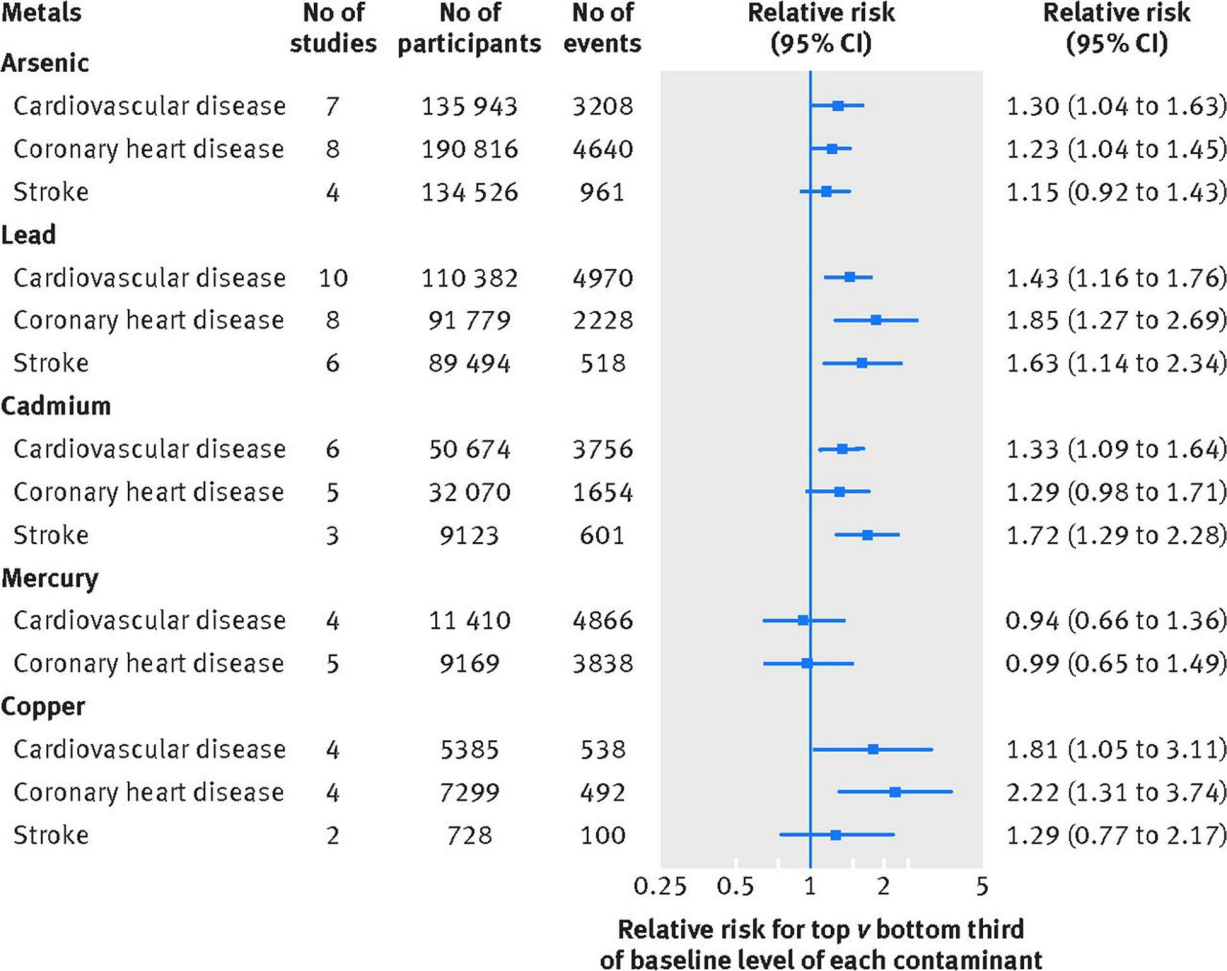
Summary of the association of environmental contaminants with cardiovascular outcomes. Pooled risk estimates were calculated using random effects meta-analyses. the relative risk compares the risk for each outcome in individuals in the top third with those in the bottom third of baseline levels of the environmental contaminants (i.e., extreme thirds). risk estimates from separate studies were typically adjusted for basic demographics (e.g., age, sex, systolic blood pressure, smoking, history of diabetes, etc.) [7]

Similarly, substantial epidemiologic evidence shows that environmental exposures to xenobiotic metal pollutants such as cadmium, lead, and arsenic are associated with increased late risks of CVD, including accelerated atherosclerotic CVD and hypertension [6]. Indeed, a large meta-analysis by Chowdhury et al. [7], including 37 studies and 348,000 participants, demonstrated that several metal contaminants (cadmium, arsenic, and lead) have a positive and direct linear dose–response with greater CVD risk (Fig. 1). For example, higher levels of cadmium were associated with a significantly increased risk of CVD, coronary heart disease, and cerebrovascular disease (relative risks [95% CI]: 1.33 (1.04–1.63); 1.29 (0.98–1.71), and 1.72 (1.29–2.28), respectively, with risk estimates based on large numbers of events (ranging from 601–3,756) and adjusted for basic demographics and CVD risk factors (age, sex, systolic blood pressure, smoking, history of diabetes, etc.). Hypothetical mechanisms of the toxicity of these metals focus on oxidative stress, lipid peroxidation, and endothelial dysfunction [6], not dissimilar to the postulated mechanisms of platinum vascular toxicity [1–3]. In particular, cadmium and lead, which can persist for decades in the human body similar to platinum [8], have been linked with increased risks of hypertension, hyperlipidemia, and atherosclerosis [6] via effects on blood pressure control, carbohydrate and lipid metabolism, vascular function, and atherogenesis [9].

Platinum-based treatment regimens for testicular cancer are a paradigm of success as curable systemic chemotherapies which result in high survival rates. However, despite success in testicular cancer survivorship, CVD may present an unintended risk of early- and late- vascular side effects of platinum-based therapies. We highlight for the first time the similarities between platinum-associated vascular adverse events and the vascular toxicity associated with other heavy-metal contaminants. The vascular toxicity seen in large epidemiologic studies of TCS may in part be similar and possibly mechanistically linked to the risk seen in environmental heavy metal contaminants linked to CVD. However, the therapeutic exposure levels of platinum in testicular cancer regimens are at much higher, curative doses than occupational or environmental exposures for other toxic heavy metals, leading us to conclude that the cardiovascular damage due to platinum may be more substantial.

Future research should be directed to better understand the magnitude of the adverse cardiovascular effects of platinum and to elucidate the underlying mechanisms of action, such as the role of epigenetic biomarkers that are induced by heavy metal exposure [10]. In the interim, following cisplatin-based chemotherapy for testicular cancer, health care providers should monitor and manage modifiable cardiovascular risk factors, including blood pressure, lipids, and glucose. Patients should also be counseled to adopt healthy behaviors, including increased physical activity, improvements in diet, smoking cessation, and the maintenance of optimal weight.

## Data Availability

None.
